# Improving Current Knowledge on Seroprevalence and Genetic Characterization of Swine Influenza Virus in Croatian Pig Farms: A Retrospective Study

**DOI:** 10.3390/pathogens10111527

**Published:** 2021-11-22

**Authors:** Andreja Jungić, Vladimir Savić, Josip Madić, Ljubo Barbić, Besi Roić, Dragan Brnić, Jelena Prpić, Lorena Jemeršić, Dinko Novosel

**Affiliations:** 1 Department of Virology, Croatian Veterinary Institute, Savska Cesta 143, 10000 Zagreb, Croatia; bsroic@gmail.com (B.R.); brnic@veinst.hr (D.B.); balatinec@veinst.hr (J.P.); jemersic@veinst.hr (L.J.); 2 Poultry Center, Croatian Veterinary Institute, Heinzelova 55, 10000 Zagreb, Croatia; v_savic@veinst.hr; 3 Deparment of Microbiology and Infectious Diseases with Clinic, Faculty of Veterinary Medicine, University of Zagreb, 10000 Zagreb, Croatia; jmadic@vef.hr (J.M.); ljubo.barbic@vef.hr (L.B.); 4 Department of Pathology, Croatian Veterinary Institute, Savska Cesta 143, 10000 Zagreb, Croatia

**Keywords:** pigs, swine influenza virus, seroprevalence, H1N1, Croatia

## Abstract

In a total of 1536 blood serum samples analysed by ELISA, antibodies for IAV nucleoprotein (NP) were detected in 30.3%. Results from HI show that the most common subtype of swIAV in the Croatian pig population was H1N1 (44.6%), followed by H3N2 (42.7%) and H1N2 (26.3%). Antibodies to at least one subtype were detected in 62.19% of blood serum samples. Detection of swIAV antigen was performed by IHC and detected in 8 of 28 lung samples collected post-mortem. The matrix (M) gene was detected in nine of one hundred and forty-two lung tissue samples and in seven of twenty-nine nasopharyngeal swabs. Phylogenetic analysis of amplified HA and NA gene fragments in Croatian isolates suggests the presence of swIAV H1avN1av.

## 1. Introduction

Swine influenza (SI) is one of the major viral diseases of pigs caused by influenza A viruses (IAVs). Although the morbidity rate in infected swine herds can be as high as 100%, the mortality rate of primary infections with swine influenza A viruses (swIAV) is very low and their involvement as important coinfection with other pathogens can cause a high mortality rate, especially in young pigs. The nature of the disease is always difficult when it is associated with other viral pathogens such as porcine respiratory coronavirus (PRCV), porcine reproductive and respiratory syndrome virus (PRRSV), the porcine circovirus type 2 (PCV2) or bacteria that can cause secondary infections such as *Actinobacillus pleuropneumoniae*, *Pasteurella multocida*, *Haemophilus parasuis* and *Streptococcus suis* type 2 [1,2,3,4]. Reproductive disorders associated with the occurrence of SI are sometimes reported by pig producers, but these are probably caused indirectly by fever and proinflammatory cytokines leading to hormonal imbalance, as well as coinfection with concurent pathogens [5,6].

The IAV genome consists of eight single-stranded viral RNA segments, each encodes at least one protein [7]. IAVs belong to the *Orthomyxoviridae* family and are characterised by the subtype of the two major surface glycoproteins, hemagglutinin (HA) and neuraminidase (NA). The most common subtypes of swIAV circulating throughout the world are H1N1, H1N2 and H3N2, but distribution of various subtypes and genotypes varies in different geographic regions [8]. The natural reservoir for IAVs is waterfowl, but humans and pigs are also considered hosts.

There are two main mechanisms by which IAVs change: antigenic drift and antigenic shift. Antigenic drift involves amino acid substitutions in the surface glycoproteins HA and NA, resulting in the appearance of different strains of virus subtypes that are, however, antigenically related and recognized by the immune system. When this accumulation occurs over a prolonged period of time, a subtype can emerge that is distinctly different from its original ancestor [9]. Antigenic shift is an exchange of RNA segments from IAVs of different origins that co-infect a single cell [10,11]. The theory of a pig as a “mixing vessel” is well known because of its susceptibility to infection with IAVs of different origin that sometimes can break species barrier with unpredictable consequences [12]. The emergence of latest pandemic IAV strain in 2009 was likely a result of a series of reassortment events in mammals over a period of years before pandemic recognition [13].

Data collected between 2010 and 2013 as part of the European Surveillance Network for Influenza in Pigs (ESNIP 3) in 16 European countries and Israel showed that swIAV was detected in 31% of the pig herds sampled. The predominant subtypes were the three European enzootic swIAV: avian-like swine H1N1 (53.6%), human-like reassortant swine H1N2 (13%) and human-like reassortant swine H3N2 (9.1%), and pandemic A/H1N1 2009 (H1N1pdm) virus (10.3%). A total 13.9% of the viruses were reassortants between these four lineages [14]. In the following comprehensive review, a group of authors reported the results of a surveillance of swIAVs conducted from April 2015 to January 2018 in 17 European countries and included 18,313 individual pig samples from 2457 farms. The prevalence of the four major swIAV lineages remained stable, but the number of swIAV reassortants carrying at least one internal genome segment (IGS) of H1pdm origin increased compared to ESNIP3 results, where most reported swIAV reassortants carried all six IGSs of the human pdm virus lineage, while less than 1% of reassortants had a mixture of avian (av) and pdm IGSs. H1avN1av was still the predominant subtype in European pig farms with a prevalence of 39.2% within the H1av lineage prevalence of 54.2%. H1hu subtypes were detected in 12.6% of farms, followed by H1pdm subtypes with 9.1% and the H3 subtype with 3.9%. Within all subtypes, reassortants were identified [15,16].

Although a high seroprevalence of swIAV in pigs and wild boar has been previously identified and the presence of the swIAV has been analysed worldwide, no similar studies have been conducted in Croatia. Only basic seroprevalence studies based on the use of commercial enzyme-linked immunosorbent assays (ELISA) and haemagglutination inhibition (HI) for detection of influenza virus antibodies in pigs and wild boar were performed, while virus isolation and identification were never performed. In 2010, the authors reported that no H1N1 antibodies were detected in pigs from large production units, but antibodies to H1N1 subtype were found in 9.23% of pigs from backyard farms [17]. One year later, 14.2% of pigs were positive for antibodies against the H1N1 subtype and 6.5% for the H3N2 subtype [18].

In 1999–2000, Croatian authors tested wild boars for antibodies against human IV and detected 18.81% positive sera for influenza virus type A H1N1, 87.12% for subtypes H3N2 and 67.32% for influenza virus type B [19]. During the two hunting seasons in Croatia, swIAV antibodies were detected in 8.6% and 11.7% of wild boars in 2005–2006 and 2009–2010, respectively [20].

The aim of this research was to complete the previous data and to investigate the prevalence of swIAV in the pig population over a period of six years through passive surveillance in eleven Croatian counties, using different diagnostic tools to detect antibodies, antigen and viral genome.

## 2. Results

### 2.1. Serology

In this study, 1536 samples of pig blood sera in the area of 11 Croatian counties were tested by ELISA for the detection of antibodies against the nucleoprotein (NP) of IAV. Antibodies against NP IAV were detected in 30.3% (95% CI 28.09–32.69) of blood serum samples. Most of the tested serum samples from pigs kept in large farms were from Osijek–Baranja (*n* = 585) and Vukovar–Srijem (*n* = 468) counties, where the pig industry is most developed. The highest IAV seroprevalence was recorded in Osijek–Baranja County where seroprevalence was estimated to be 100% in F3 farm, followed by F4 (71.4%) and F9 (68.9%) farms. A slightly lower mean seroprevalence of 70% was recorded in the farms at Virovitica–Podravina County. In pigs kept in backyard farms in the continental part of Croatia, the highest mean seroprevalence of 89.7% was also found on Brod–Posavina County, while no positive sample was found in backyard farms on Zagreb County. Seroprevalence of swIAV in backyard farms in the Croatian coastal region was lowest in Šibenik–Knin County 15.2%, followed by Istria 44,4% and Dubrovnik–Neretva County 45.5%. H1N1 subtype was detected in almost all farms, except in farms F5 and F10 in Osijek–Baranja County and in farm F17 in Varaždin County, where none of the subtypes was detected. H3N2 subtype was not detected in backyard farms in Karlovac, Šibenik–Knin and Zagreb County counties, as well as H1N2 subtype, which was not detected in nine counties. Seroprevalence of IAV with a 95% confidence interval is shown in Table 1.

Among the pig categories, the lowest seroprevalence was found in 5–10 week old pigs and boars (12.7% and 12.9%, respectively), while more than half of the sera tested from fatteners and first parity sows (56.2% and 61.4%, respectively) were positive for IAV nucleoprotein (NP) antibodies (Table S1).

Because of the small volume of sera delivered to the laboratory and other analyses that had to be performed, only 410 of the total 466 NP antibody-positive sera were tested by HI with reference swIAV antigens for three strains: avian-like H1N1, triple reassortant H1N2, and avian-like H3N2. Antibodies for at least one swIAV subtype were detected in 62.19% of positive samples (*n* = 255). Nearly evenly represented were H1N1 (44.6%) and H3N2 (42.7%) subtypes, followed by H1N2 (26.3%). As a result of coinfection with different swIAV, antibodies against two or three swIAV subtypes were detected in individual categories of pigs (Table 2 and Table 3). Mean seroprevalence of IAV positive serum samples subtyped by HI (%) presented in different age groups is shown in Table S2.

### 2.2. Immunohistochemistry (IHC)

**Figure 1:**
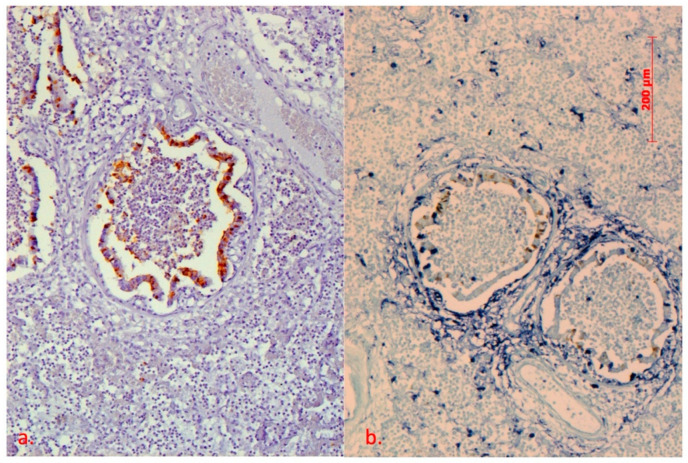
Lungs. (**a**) IHC to detect H1N1/H3N2, DAB chromogen, counterstain with Mayer’s heamatoxylin, (**b**) IHC to detect H1N1/H3N2, DAB chromogen, ISH to detect PCV2, NBT chromogen, counterstain with FastGreen 10X. Severe mononuclear infiltration in alveolar spaces, almost no air present. Desquamation of the ciliary epithelial layer in the bronchi, while the lumen is filled with inflammatory cells and necrotic cell debris. A strong positive SIV antigen signal was present in the epithelial cells in the cytoplasm and nuclei, partially in the cells in the lumen of the bronchi and in a few cells in the lung parenchyma. Positive PCV2 genomic signal was present in peri-bronchial connective tissue, cells in the alveolar walls, ciliary epithelial cells, and necrotic debris in the bronchial lumen.

**Figure 2:**
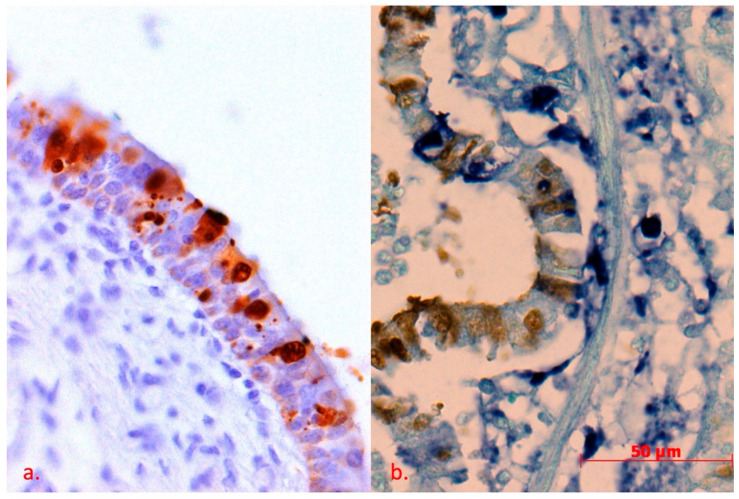
(**a**) Cross-section through the epithelial layer of the bronchi. IHC to detect H1N1/H3N2, DAB chromogen, counterstain with Mayer’s hematoxylin, (**b**) IHC to detect H1N1/H3N2, DAB chromogen, ISH to detect PCV2, NBT chromogen, counterstain with FastGreen 40X. A different grade of apoptosis of ciliated epithelial cells are visible. All cells that are apoptotic are strongly positive for H1N1/H3N2 antigen. In particular, a strong aggregation of the antigen into granules in the cytoplasm is seen as a result of cariolysis. Some of the H1N1/H3N2 positive cells are still viable and antigen can be observed in the cytoplasm and nuclei. Most of the cells were infected with SIV only. In some cells there was only PCV2. In doubly infected cells, signals for both viruses can be found in the nuclei and cytoplasm. Not surprisingly, these cells are severely damaged and lack normal cell structure.

Of a total of twenty-eight lung samples examined, eight were positive for swIAV antigen. Of these, five were from 9–14-week-old piglets (F14) and three were from 4–10 week old piglets (F13) from Vukovar–Srijem County (Figure 1a and Figure 2a). From the backyard farm Brod–Posavina County 3 samples of lung tissue from piglets aged 9–12 weeks were examined but no swIAV antigen was detected. Of the eight positive lung samples for swIAV antigen from F14 in Vukovar–Srijem County, two were subjected to double detection/staining: in situ hybridization (ISH) to detect PCV2 and immunohistochemistry (IHC) to detect swIAV, as the animals were affected by postweaning multisystemic wasting syndrome (PMWS) (Figure 1b and Figure 2b).

### 2.3. M Gene Detection

Viral RNA was detected in nine of a total one hundred and forty-two lung samples and in seven of total twenty-nine nasopharyngeal swabs. swIAV was detected in 4–10-week-old piglets in farm F13 (*n* = 10) and in farm F14 (*n* = 5) at Vukovar–Srijem County. One positive sample was from an approximately 10–12-week-old piglet from a backyard farm in Brod–Posavina County. Number of total lungs tested by real-time RT-qPCR (M gene) and quantification cycle (Cq) values are presented in Tables S3 and S4.

### 2.4. swIAV Subtyping

HA and/or NA gene was partially amplified in five of nine swIAV-positive lung samples and in three of seven swIAV-positive nasopgaringeal samples. swIAV-positive lung samples from Vukovar–Srijem County were obtained from 9–14 weeks old piglets from farm F14 numbered 2319/4/2011 and 1600/9/2012 (hereafter 2319/2011 and 1600/2012) and from 4–10 weeks old piglets from farm F13 numbered 854/5/2016 and 855/4/2016 (hereafter 854 and 855/2016). In the lung sample of a 10–12 weeks old piglet from a backyard farm in Brod–Posavina County, numbered 631/3/2015, only the coding HA2 region of the HA gene was amplified, but it was not included in the philogenetic analysis due to poor sequence quality. The HA and NA genes were partially amplified in 3 nasopharyngeal swabs from 4–6 weeks old piglets numbered 1/2016, 3/2016 and 5/2016, obtained from farm F13 in Vukovar–Srijem County.

Analyses of the amplified HA1 and HA2 gene segments with an expected size of ~327 bp and ~640 bp, respectively, and the NA gene segment of ~514 bp indicate that the isolates belong to the avian-like H1 and N1 swIAV.

### 2.5. Phylogenetic Analysis

Two partial sequences of the HA gene (HA1 and HA2 domains) and one partial sequence of the NA gene were analysed. Based on two different coding regions and sizes of the analysed sequences, two phylogenetic trees of the HA gene were constructed. The sequences were deposited in GenBank under the following accession numbers: OK070751, OK189574 (A/sw/Croatia/2319/2011); OK189565, OK070753, OK189573 (A/sw/Croatia/1600/2012); OK070749 (A/sw/Croatia/631/2015); OK189572 (A/sw/Croatia/1/2016); OK189566, OK189575 (A/sw/Croatia/3/2016); OK189567 (A/sw/Croatia/5/2016); OK189568, OK070750, OK189576 (A/sw/Croatia/854/2016); OK070748 (A/sw/Croatia/855/2016).

All Croatian isolates belong to the Eurasian avian-like H1N1 lineage according to the two analyses: Most common recent ancestor (MRCA) tree and Median-joining (MJ) networks. According to the HA gene sequences, they are all clustered in clade C1 [21].

**Figure 3:**
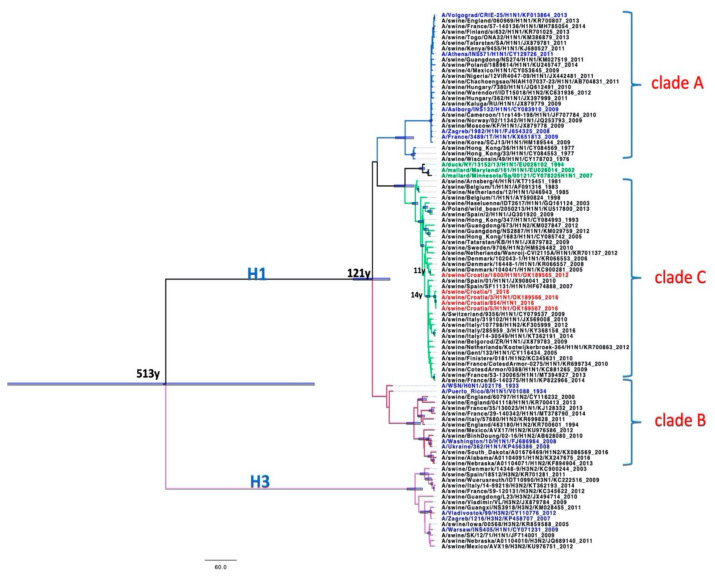
MRCA tree of HA1 gene. Blue bars represent clade credibility interval where posterior probability >0.9. Taxa names were coloured: red—Croatian sequences, black-isolates from pigs, blue—isolates from humans, green—isolates from birds. Scale bar represents 60y.

**Figure 4:**
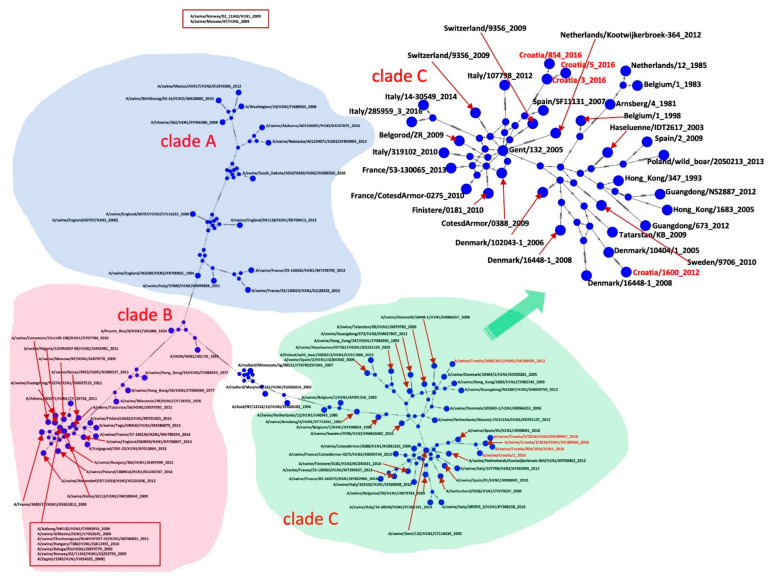
MJ networks of HA1 gene. Blue dots represent haplotypes. Croatian sequences have taxa names coloured in red. Hatch marks represent mutations.

**Figure 5:**
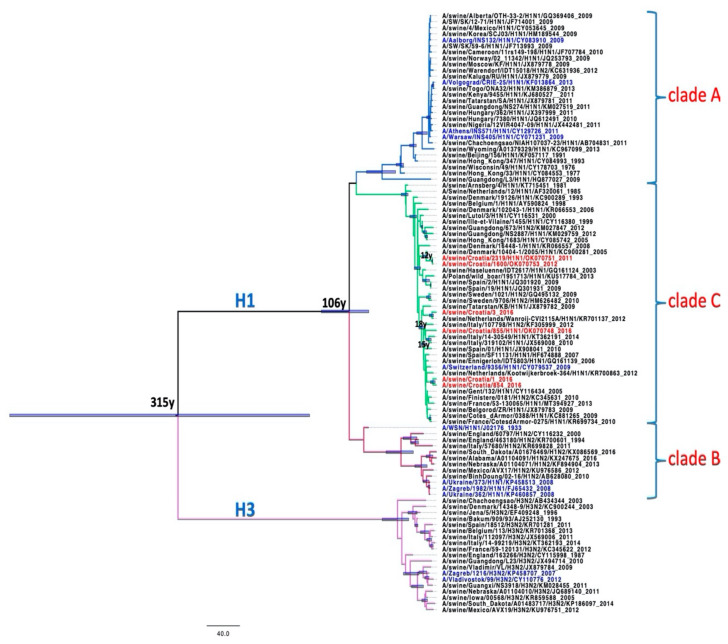
MRCA tree of HA2 gene. Blue bars represent clade credibility interval where posterior probability >0.9. Taxa names were coloured: red—Croatian sequences, black-isolates from pigs, blue—isolates from humans. Scale bar represents 40y.

**Figure 6:**
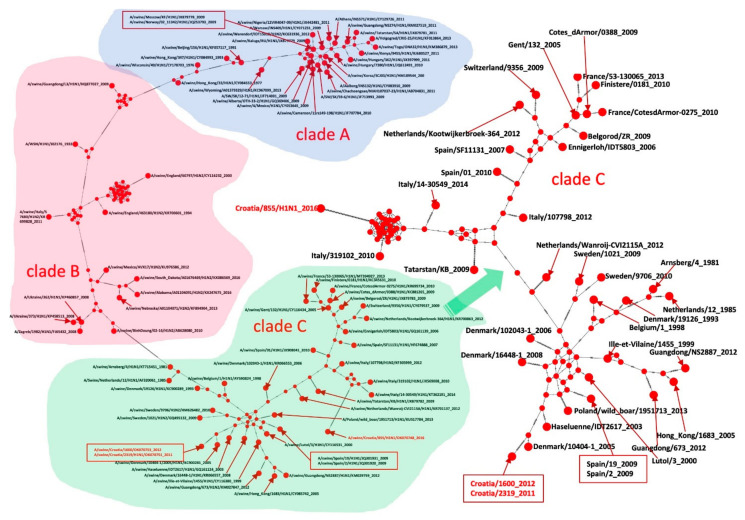
MJ networks of HA2 gene. Red dots represent haplotypes. Croatian sequences have taxa names coloured in red. Hatch marks represents mutations.

Sequences A/sw/Croatia/2319 and A/sw/Croatia/1600 from 2011 and 2016 appear retrospectively to be direct descendants of A/sw/Denmark/10404-1, which was isolated in 2005 (HA and NA). A/sw/Croatia/1, A/sw/Croatia/3, and A/sw/Croatia/854 appear to be descendants of A/sw/Spain/SF11131 from 2007 according to the HA gene (Figure 3, Figure 4, Figure 5 and Figure 6).

According to the MRCA tree of HA1 (Figure 3), Croatian sequences A/sw/Croatia/2319/2011 and A/sw/Croatia/1600/2012 appear to be direct descendants of A/sw/Denmark/10404-1, which branched around 2005 with a statistically significant posterior probability > 0.9. The Croatian sequences A/sw/Croatia/1, A/sw/Croatia/3, A/sw/Croatia/5 and A/sw/Croatia/854 from 2016 branched in the clade 14 years before with isolates from Italy, France, the Netherlands, Finistere, Belgorod, Belgium and one human isolate in Switzerland. MJ networks of HA1 (Figure 4) confirmed that A/sw/Croatia/2319/2011 and A/sw/Croatia/1600/2012 are descended from A/sw/Denmark/10404-1, while A/sw/Croatia/1, A/sw/Croatia/3, A/sw/Croatia/5 and A/sw/Croatia/854 from 2016 appear to evolve from A/sw/Spain/SF11131-2007, which evolved from A/sw/Gent/132-2005 as the ancestral strain of the entire clade. Both the MRCA tree and the MJ networks exclude that the Croatian sequences could have descended from the A/swine Haselünne/IDT2617 inoculation strain. The timing of the divergence was much earlier than the vaccine was available on the market.

The MRCA tree of HA2 reconfirms (Figure 5) that the Croatian sequences A/sw/Croatia/1600 appear to be direct descendants of A/sw/Denmark/10404-1, which branched around 2004 with a statistically significant posterior probability > 0.9. The MJ networks (Figure 6) suggest that these sequences may have A/swine Haselünne/IDT2617 as an ancestral strain, but the MRCA tree shows clear and statistically significant branching much before the branching with the Danish strain. The MRCA tree shows that A/sw/Croatia/1, A/sw/Croatia/3, A/sw/Croatia/854, and A/sw/Croatia/855 from 2016, branched together with French, Spanish, Italian, and Dutch sequences, as well as a sequence isolated from humans in Switzerland. According to MJ networks, A/sw/Croatia/855 was derived from Italian strains. The method did not allow us to calculate the topology distance for A/sw/Croatia/1, A/sw/Croatia/3 and A/sw/Croatia/854 because of gaps in the sequences.

**Figure 7:**
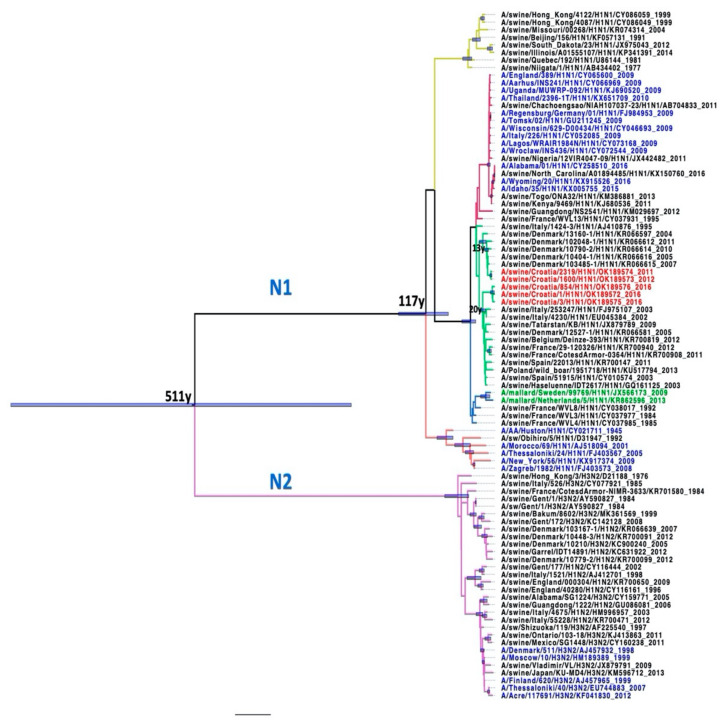
MRCA tree of NA gene. Blue bars represent Clade credibility interval where posterior probability >0.9. Taxa names were coloured: red—Croatian sequences, black-isolates from pigs, blue—isolates from humans, green—isolates from birds. Scale bar represents 60y.

**Figure 8:**
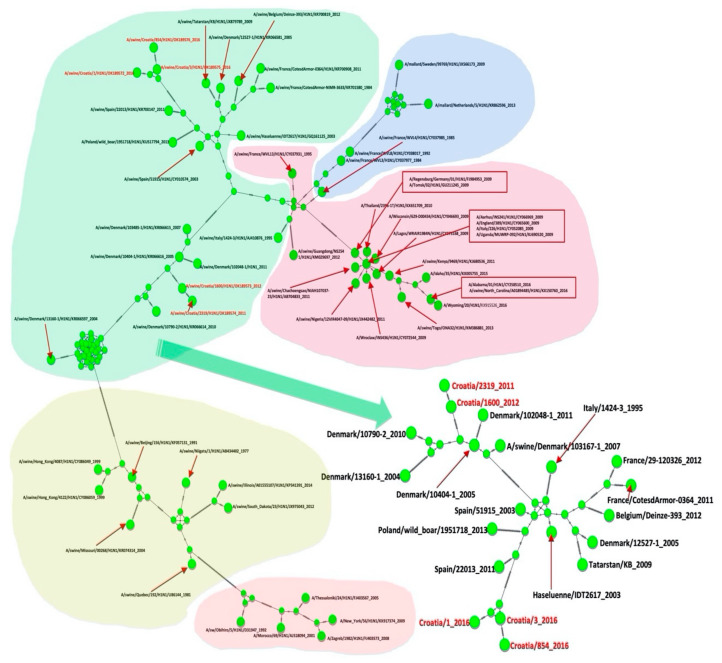
MJ networks of NA gene. Green dots represent haplotypes. Croatian sequences have taxa names coloured in red. Hatch marks represents mutations.

The results of the MRCA tree for the NA gene (Figure 7) are consistent with the results for HA1 and HA2. The Croatian sequences A/sw/Croatia/1600/2012 and A/sw/Croatia/2319/2011 appear to be direct descendants of A/sw/Denmark/10404-1, which branched around 2003 with a statistically significant posterior probability > 0.9, which also supports the results of MJ Networks (Figure 8). Sequences A/sw/Croatia/1, A/sw/Croatia/3 and A/sw/Croatia/854 were in a clade together with the Italian and French sequences, the sequence Haselünne/IDT2617 and the sequence isolated from Polish wild boar. MJ networks suggest that Spanish strains may be ancestral to this Croatian branch. Sequences A/sw/Croatia/2319 and A/sw/Croatia/1600 from 2011 and 2012, respectively, appear to be retroactively direct descendants of A/sw/Denmark/10404-1, which was isolated in 2005 (HA and NA). According to NA, A/sw/Croatia/1, A/sw/Croatia/3 and A/sw/Croatia/854/5 from 2016 appear to have the same ancestors as two Spanish, Italian and French isolates, one from Germany, Belgium and Tatarstan and swIAV isolated from wild boar in Poland (Figure 7 and Figure 8).

According to both genes, Croatian sequences are phylogenetically divided into two branches. One group of sequences is related to Danish strains, while the second is related to Spanish and Italian sequences. In all two cases, the branching was performed around 2002. A/sw/Croatia/855/2016 showed the same phylogenetic relationship with Spanish, Italian and Tatarstan sequences according to the HA2 gene and belongs to the same branch as A/sw/Croatia/1/2016, A/sw/Croatia/3/2016 and A/sw/Croatia/854/5/2016.

### 2.6. Virus Isolation

Five lung samples (2319/2-4/2011, 1600/9/2012 and 631/3/2015) and seven nasopharyngeal swabs (1-7/2016) were inoculated into Madin–Darby canine kidney (MDCK) cells. Cell-passed samples were analyzed after each passage using real-time RT-qPCR to detect the M gene and to investigate whether each cell line could amplify swIAVs. All samples were subjected to a third passage, although only four of them had lower Cq levels than after two passages (2319/4, 631/3, 1 and 3/2016). In the third passage, swIAV was isolated in samples 2319/4/2011 and 1600/2012 (with higher Cq values in the 2nd than in the 3rd passage) (Figure S1).

## 3. Discussion

Swine influenza is a very common disease in pigs, and influenza viruses have been identified as one of the most frequently isolated pathogens in outbreaks of acute respiratory disease in pigs. Therefore, there was a lack of data on circulating swIAV strains in Croatia and neighbouring countries of Southeast Europe during this research. Croatian livestock began to recover after the war, and between 2004 and 2007 increased imports of reproductive animals, including gilts, but the total number of pigs in Croatia still barely exceeds 1 million per year (data from EU-COMTRADE (http://comtrade.un.org/, accessed 18 October 2020) and the Croatian Agricultural Agency (https://hpa.mps.hr/, accessed 18 October 2020). Croatia’s accession to the EU in 2013 has not led to the expected increase in domestic pig production, so imports of pigs are increasing, as Croatian pig production cannot meet consumption [22]. These data suggest that international trade may be one of the possible transmission routes for swIAVs which is consistent with previous studies on the importation of new strains of PCV 2 and PRRSV, two other important viral pathogens of pigs in Croatia [23]. Large pig farms are located in the continental part of Croatia, especially in Osijek–Baranja and Vukovar–Srijem counties, which were included in this study. Small backyard farms are still maintained, but the number of pigs kept for home consumption is rapidly increasing.

The aim of this study was to identify and compare the results of previous studies in Europe and to describe the seroprevalence and basic genetic characteristics of swIAV in Croatia.

The seroprevalence of swIAV in Croatia was based on individual animal testing and estimated at 30.3%. As expected, seroprevalence and incidence of each subtype varied from one farm to another. It is not uncommon for pigs to have concurrent antibodies to different swIAV subtypes, either due to contact with the virus during life or as a result of maternally derived antibodies (MDA) [24]. The dominant subtypes in the Croatian pig population were H1avN1 and H3N2 (44.6% and 42.7%) followed by H1huN2 (26.3%). It was found that 71.4% of fatteners, 73.7% of boars and 54.8% of pigs of unknown category (mostly from backyard farms) reacted negatively to swine specific IAV antigens used in the study, although they had antibodies against NP of IAV. In gilts and 4–10 weeks old piglets, only 6.8% and 12.9% of the subtypes are unidentified, respectively, which may indicate persistent infection with a specific swIAV subtype in herd. An increased percentage of samples from 11–20-week-old fatteners with unidentified antibody subtype could indicate the introduction of swIAV from another farm or country, as they are usually imported at 10–12 weeks of age. Antibodies against a conserved influenza A nucleoprotein epitope react with numerous influenza A subtypes from different species and are induced earlier than to hemagglutinins. Significantly, this ELISA format can detect the antibodies of all test antigen-specific classes of immunoglobulins (Ig) [25,26]. In contrast, HI is subtype specific and due to the diversity of circulating swIAVs, it is more difficult to obtain a satisfactory result. Since data from previous studies indicate that the H1N1pdm strain is becoming enzootic in many European countries [14,16] it is likely that it was introduced into Croatian pig herds at some point, but has not been detected. The fact that different subtypes of swIAV persist and circulate in herds and that human IAVs can be transmitted to pigs increases the possibility of the emergence of new reassortant swIAVs. Therefore, antibodies against at least one subtype of the virus were detected in all herds except Zagreb County in a farm with high biosecurity conditions and backyard farms. Maintaining a high level of biosecurity goes a long way in preventing the introduction of influenza viruses into the pig herd, but in a small backyard farms it is difficult to avoid pig-associated interspecies contacts, which pose a significant risk for interspecies transmission of influenza viruses [27,28].

The introduction of a new strain of swIAV into a herd is thought to be associated with clinical signs of disease in a large number of animals. The frequency of confirmed outbreaks with clinical signs of disease in pigs is relatively low, and the infection is thought to occur much more frequently in a subclinical form [29,30]. If we exclude the classical outbreak of SI, there is very little knowledge about the dynamics and maintenance of endemic SI. Suckling and weaning piglets are among the most susceptible age groups to influenza infection because antibodies against the homologous swIAV strain do not fully protect them from a new infection with the same or a similar strain [31,32].

In this study, pigs of different age were examined for the presence of swIAV, but only piglets aged 4 to 14 weeks were positive for swIAV and some of them developed severe clinical signs of respiratory disease. The first detection of swIAV antigen in the lungs of Croatian pigs was obtained in this study by an immunohistochemical procedure in piglets suffering primarily from post-weaning multisystemic wasting syndrome (PMWS), a disease associated in part with porcine circovirus (PCV). Simultaneous infection with swIAV and PCV2 leading to the increased piglet mortality was detected by IHC/ISH and confirmed by molecular methods.

Considering the low seroprevalence in younger pig groups and the relatively low antibody titer, we can conclude that piglets did not have adequate protection against swIAV infection. A very high antibody titer to H3N2 subtype (≤2048) was detected in first parity sows, and a titer for H1N1 and H1N2 subtypes of ≤512 was equally detected in sows and first parity sows (Table 3). Therefore, it is more likely that they have been exposed to the swIAV at some point in their lives, but the role of sows in maintaining swIAV circulation in commercial farms in Croatia is also still unknown and requires further research.

Vaccination of pigs against swine influenza was not performed in Croatian pig population during this study, so the antibody titre indicates the exposure of animals to infection with a particular subtype. The use of additional reference swIAV antigens, including that for the pandemic virus subtype, would provide us a better insight into the occurrence of new strains in the pig population.

Euroasian avian-like swine H1N1 viruses are the predominant viruses in the pig population in Europe, but an increasing number of reassortments between the predominant enzootic swIAVs and the H1N1pdm have been observed [33,34,35,36,37]. The enzootic H1avN1av lineage is present in most European countries and usually circulates together with reassortant strains. In pig herds in countries with advanced pig production, reassortants of H1N1 and H1N2 are reported more frequently than H3N2 reassortants [38,39]. The sequences of Croatian isolates show a strong phylogenetic relationship with European strains of H1avN1av and according to Bayesian inference, they were rooted before 2002, 14 years before the last Croatian isolate. The phylogenetic analysis was based only on the nucleotide sequences of HA1 and HA2 and NA gene segments of the isolated viruses, so it was not possible to determine to which genotype the isolates from this study belong without analysis of internal segments of swIAV. The absence of subtyped isolates, despite the detection of a highly conserved region of the IAV matrix gene, supports the conclusions of the serological investigation.

This study provided updated knowledge on the swine influenza viruses in the Croatian pig population, but continuous virological surveillance is needed to better understand the evolution of influenza viruses and the role of pigs in the IAVs ecology.

## 4. Materials and Methods

### 4.1. Samples

Blood samples were collected from pigs of different categories (piglets, weaners, fatteners, gilts, sows, boars) and production units (commercial farms and backyard farms) during National Surveillance Programme of Aujeszky’s disease in domestic pigs in Croatia. A small part of blood samples was collected during routine diagnostic procedures on Croatian Veterinary Institute, Virology Department. Between 2011 and 2016, a total of 1536 blood samples from 17 large production units (400–2000 pigs) and 8 backyard farms were tested for the presence of swIAV antibodies.

**Figure 9:**
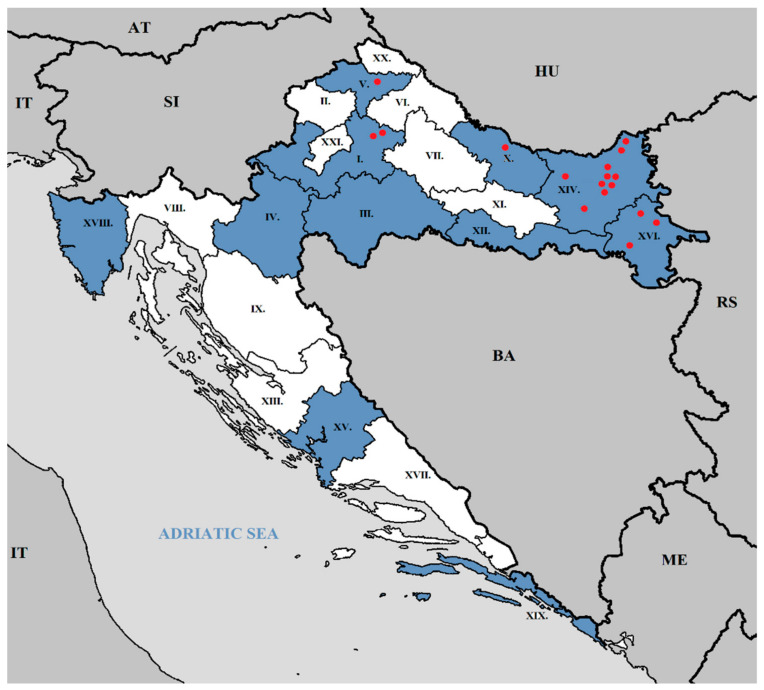
Map of Croatia with counties. I. Zagreb County, II. Krapina–Zagorje, III. Sisak–Moslavina, IV. Karlovac, V. Varaždin, VI. Koprivnica–Križevci, VII. Bjelovar–Bilogora, VIII. Primorje–Gorski Kotar, IX. Lika–Senj, X. Virovitica–Podravina, XI. Požega–Slavonia, XII. Brod–Posavina, XIII. Zadar County, XIV. Osijek–Baranja, XV. Šibenik–Knin, XVI. Vukovar–Srijem, XVII. Split–Dalmatia, XVIII. Istria, XIX. Dubrovnik–Neretva, XX. Međimurje, XXI. The City of Zagreb County. Blue color: examined counties. Red dots: examined farm (F1-17) locations.

Lung samples were collected from pig carcasses with evidence of broncho-interstitial pneumonia submitted to the Laboratory for Pathological Morphology at Croatian Veterinary Institute for necropsy, and at the slaughterhouse. Nasopharyngeal swabs were collected from pigs with signs of respiratory disease by the authorized Veterinary Service for each farm. None of the animals had been vaccinated against swIAV during this study. Counties included in study are presented in Figure 9.

### 4.2. Serology

Blood sera were first analysed by ELISA for the detection of anti-influenza A virus nucleoprotein (NP) antibodies (ID Screen^®^ Influenza A antibody competition multispecies, ID.vet, Grabels, France) according to the manufacturer’s instructions. Internal positive and negative controls were within the limits recommended in the manufacturer’s instructions. The absorbance was measured at 450 nm using a microplate reader (TECAN, Austria). Samples with a serum to negative control competition percentage (S/N%) greater than or equal to 50% were classified as negative and samples with S/N% less than or equal to 45% were classified as positive. Samples with S/N% between 45% and 50% were classifies as doubtful and retested. Depending on the S/N% obtained in the second test, samples were classified as either negative (S/N% > 50) or positive (S/N% < 50). Sera that tested positive by ELISA were further analysed using a hemagglutination inhibition assay (HI) with reference antigens A/swine/Neth/Best/96 (H1avN1), A/swine/Gent/7625/99 (H1huN2) and A/swine/Neth/St Oedenrode/96 (H3N2), provided by GD Animal Health, Deventer, The Netherlands. For the quality control, H1N1 and H3N2 positive sera from the same provider were included in each run. Before the HI test, nonspecific serum hemagglutination inhibitors were removed with receptor-destroying enzyme from Vibrio cholerae (Denka Seiken Co., Ltd., Tokyo, Japan). The HI assay was performed according to standard procedures [40], using 1% chicken red blood cells (RBCs) and 4 hemagglutinating units of each virus. HI antibody titre of each sample was determined as the reciprocal of the highest dilution at which no hemagglutination was observed. A result of ≥1:16 (24) was considered indicative of positive exposure status for a specific strain.

### 4.3. Immunohistochemistry

Immunohistochemical labelling of swIAV antigen was performed using a specific mouse monoclonal antibody (Mab) against H1N1/H3N2 (Ingenasa^®^ Immunologia y Genetica Aplicada, S.A., Madrid, Spain). Sections were deparaffinized in xylene and rehydrated with different grades of ethanol. Endogenous peroxidase was blocked with 3% H202 in 0.5 M/0.15 M Tris/NaCl (TBS). For detection of swIAV, tissue sections were simultaneously enzymatically pre-treated with proteinase K, 40 µL proteinase K in 2 mL TBS (according to the manufacturer’s instructions) for 3 min at RT. To avoid non-specific reactions, the tissue was blocked with 2% bovine serum albumin (BSA) in TBS for 1 h. The primary antibody was diluted 1:200 in 2% BSA in TBS and incubated overnight at room temperature. The reaction was visualized with a secondary anti-mouse antibody labelled with a labelling polymer (Dako EnVision, Santa Clara, CA, USA) according to the manufacturer’s instructions. For visualization of the reaction, DAB chromogen was used, slides were counterstained with Mayer’s haematoxylin and then dehydrated and mounted with DPX.

In two lung samples where coinfection with PCV2 was detected, paraffin blocks were subjected to double detection/staining ISH to detect PCV2 and IHC to detect swIAV.

The ISH procedure for detection of PCV2 was performed as previously described [41]. Briefly, 3 μm thick tissue sections were cut and placed on silanized slides. After deparaffinization and rehydration of the tissue sections, enzymatic pretreatment with 0.3% pepsin was performed for 10 min at 37 °C and another 8 min during heating to 105 °C. The sections were then incubated at the latter temperature with 100% formamide for 5 min. A pre-hybridization step was performed by incubation with the corresponding specific probe at 105 °C for 5 min, followed by a hybridization step at 37 °C for 60 min. The detection system consisted of an anti-digoxigenin antibody conjugated to alkaline phosphatase and the substrate NBT/X-Phos (nitro-blue tetrazolium/5-bromo-4-chloro-3-indolepfosphates). To obtain a double staining of ISH for the detection of PCV2 and IHC for the detection of swIAV according to ISH procedure of colour development, the slides were immersed in 0.1 M Tris-buffered saline (TBS) without counterstaining and then IHC was performed without enzymatic pretreatment and with blocking of endogenous peroxidase [42]. The slides were finally counterstained with FastGreen.

### 4.4. Molecular Methods

#### 4.4.1. RNA Extraction

Viral RNA was extracted in iPrep™ Purification Instrument using isolation kit PureLink TM Virus Kit (InvitrogenTM/Thermo Fisher Scientific, Carlsbad, CA, USA) according to the manufacturer’s instructions. The extracted RNA was stored at −80 °C until testing.

#### 4.4.2. Real-Time RT-qPCR

Detection of swIAV in nasopharyngeal swabs and lung tissues was performed using quantitative real-time reverse-transcriptase polymerase chain reaction (RT-qPCR) to detect the M gene of influenza A viruses in a QPCR system, Stratagene Mx3005P (Agilent Technologies, Santa Clara, CA, USA). This study used the protocol established by the WHO Collaborating Centre for influenza at the CDC (Atlanta, GA, USA) [43] and a primer and probe set designed for universal detection of IAVs. All samples were analysed for the presence of the mammalian beta-actin mRNA [44]. Detection of this reference gene served both as an internal control and to check for the presence of nucleic acids.

The sample was considered positive if the reaction growth curve crossed the quantification cycle line (Cq) within 40 cycles.

#### 4.4.3. RT-PCR

IAV real-time RT-qPCR positive samples were subtyped using the multiplex RT-PCR described by Chiapponi et al. [45] for the detection of H1, H3, N1 and N2 genes. For detection of the conserved region of the HA2 gene of influenza A subtypes, we used a RT -PCR described by Phipps et al. [46]. One-step reverse transcription and amplification were performed in Thermocycler Gene Amp PCR System 9700 (Applied Biosystems, Waltham, MA, USA) using SuperScript ™ III One-Step RT -PCR System Kit with Platinum ™ Taq DNA Polymerase (Invitrogen, Carlsbad, CA, USA) according to the manufacturer’s instructions. PCR products were separated by gel electrophoresis using a 1.5% (*w*/*v*) agarose gel in Tris-borate EDTA buffer, stained with GelStar™ Nucleic Acid Gel Stain (Lonza) and visualised under UV light (Vilber Lourmat, Torcy, France). PCR products were purified (Wizard SV Gel and PCR Clean-Up System (Promega, Madison, WI, USA) and sequenced by Macrogen Inc. (Amsterdam, The Netherlands).

#### 4.4.4. Sequence Analysis

Two different methods were used for phylogenetic analysis:

(a) Bayesian inference using BEAST 1.10.4. to analyse the evolutionary background of the Croatian isolates and to analyse the haplotype diversity and for better illustration and (b) median joining network was calculated.

Sequences were aligned using the program ClustalW implemented in MEGA 7 software to create a. nexus file. Three different datasets were analysed (i) 97 sequences, 287 bp, from 86–372 nt of the hemagglutinin region HA1 encoding the HA gene (ii) 96 sequences, 595 bp length, 1107–1701 nt of the hemagglutinin region HA2 encoding the HA gene (iii) 91 sequences, 522 bp length, 617–1138 nt encoding the NA gene. The sequences were selected using the BLAST tool, randomly selecting the sequences that were highly similar and the others that were less related and represented different clades. Sequences were downloaded from the Influenza Research Database (https://www.fludb.org/ accessed on 18 October 2020) using an internationally accepted standard for naming influenza virus sequences. In addition, the years of isolation were added for tip dating in the BEAST. For the calculation of Median joining (MJ) network of the clade with the Croatian sequences, the name of the taxa, the host of origin and the year of collection were removed for better visibility, but all data are traceable. Croatian sequences that do not have a GeneBank accession number in the taxa name were incomplete and were not submitted to GeneBank. They can be used for Bayesian inference to obtain information on approximate phylogenetic relationships and do not cause errors, but not for MJ networks where the calculation fails.

The Bayesian phylogeny was calculated using BEAST1.10.4 [47]. The program was run with Markov chain Monte Carlo length until Effective sample size exceeded 200. Calculations were performed using generalize times reversible (GTR) substitution model [48], the relaxed lognormal molecular clock [49] and the Coalescent constant population size model. Tracer v1.6 software was used to analyse the results. The selected tree file was compiled in TreeAnnotator v1.10.4 from the BEAST package [47]. Clade credibility intervals or 95%HPD were calculated for rooting, where the value of posterior probability was 0.90. Most recent common ancestor (MRCA) trees were created in FigTree v1.4.3. Median joining network was calculated using the same datasets, removing H3N2 sequences in the first step, since all emerging sequences belong to the H1N1 subtype, and only clades with Croatian sequences in the next step. Sequence polymorphism was calculated using DnaSP v6 software [50] and a median joining (MJ) network was calculated using PopArt software [51].

### 4.5. Virus Isolation

IAV positive samples detected by IHC and/or Real-time RT-qPCR were prepared and inoculated into Madin–Darby canine kidney (MDCK) cell line as described before [52]. Flasks (T-25, NUNC, Rochester, NY, USA) were incubated at 37 °C/CO_2_ and cells were observed daily for cytopathic effect (CPE). Cell culture was harvested at approximately 75% (3+) of CPE observed by collecting supernatant fluid. Both samples that produced or did not produce CPE were subjected to a second passage. Culture supernatants were tested for the presence of swIAV by real-time RT-qPCR after every passage and if Cq values were lower than in previous passage it was assumed that virus replicated. A total of three passages were made.

### 4.6. Statistics

The prevalence of antibodies to swIAV was estimated with a 95% confidence interval [53].

## Figures and Tables

**Table 1:** Seroprevalence of IAV in large farms (F) and backyard farms (BF) in Croatian counties.

County	F/BF	IAV Ab ELISA		
		*n* Samples Tested	*n* Positive/Seroprevalence (%)	CI (95%)
Brod-Posavina	BF	58	52 (89.7%)	79.22–95.17
Dubrovnik–Neretva	BF	22	10 (45.5%)	26.9–65.34
Istria	BF	9	4 (44.4%)	18.87–73.33
Karlovac	BF	37	6 (16.2%)	7.65–31.14
Osijek–Baranja	F1	215	49 (22.8%)	17.69–28.85
	F2	60	22 (36.7%)	25.62–49.32
	F3	10	10 (100%)	72.25–100
	F4	35	25 (71.4%)	54.95–83.67
	F5	5	3 (60%)	23.07–88.24
	F6	30	11 (36.7%)	21.88–54.49
	F7	30	16 (53.3%)	36.14–69.76
	F8	68	7 (10.3%)	5.07–19.75
	F9	90	62 (68.9%)	58.72–77.52
	F10	40	3 (7.5%)	2.58–19.86
	BF	2	1 (50%)	n.a.
Sisak–Moslavina	BF	9	1 (11.1%)	1.99–43.5
Šibenik–Knin	BF	33	5 (15.2%)	6.65–30.92
Varaždin	F17	34	4 (11.8%)	4.67–26.62
Virovitica–Podravina	F11	10	7 (70%)	39.68–89.22
Vukovar–Srijem	F12	95	23 (24.2%)	16.71–33.72
	F13	349	134 (38.4%)	33.45–43.6
	F14	24	10 (41.7%)	24.47–61.17
Zagreb	F15	156	1 (0.6%)	0.11–3.54
	F16	105	0	0
	BF	10	0	0
Total:		1536	466 (30.3%)	28.09–32.69

**Table 2:** swIAV subtypes detected by HI in IAV Ab positive samples in farms (F) and backyard farms (BF).

County	F/BF	HI Test			
		*n* Positive Subtype/% Positive Subtype			*n* Tested Samples
		H1N1	H3N2	H1N2	
Brod–Posavina	BF	10 (17.9%)	1 (1.8%)	1 (1.8%)	56
Dubrovnik–Neretva	BF	nd			
Istria	BF	1 (25%)	1 (25%)	0	4
Karlovac	BF	4 (66.7%)	0	0	6
Osijek–Baranja	F1	34 (72.3%)	40 (85.1%)	32 (68.1%)	47
	F2	4 (18.2%)	9 (40.9%)	1 (4.5%)	22
	F3	nd			
	F4	8 (40%)	5 (25%)	0	20
	F5	0	0	0	0
	F6	1 (16.7%)	1 (16.7%)	1 (16.7%)	6
	F7	2 (18.2%)	2 (18.2%)	2 (18.2%)	11
	F8	7 (77.8%)	1 (11.1%)	0	9
	F9	16 (34.8%)	6 (13%)	6 (13%)	46
	F10	0	0	0	3
	BF	1 (100%)	1 (100%)	1 (100%)	1
Sisak–Moslavina	BF	nd			
Šibenik-Knin	BF	1 (20%)	0	0	5
Varaždin	F17	0	0	0	4
Virovitica–Podravina	F11	1 (14.3%)	7 (100%)	0	7
Vukovar–Srijem	F12	10 (41.7%)	7 (29.2%)	8 (33.3%)	24
	F13	82 (59.4%)	94 (68.1%)	55 (39.9%)	138
	F14	nd			
Zagreb	F15	1 (100%)	0	1 (100%)	1
	F16	nd			
	BF	nd			
Total:		183 (44.6%)	175 (42.7%)	108 (26.3%)	410

**Table 3:** Number of serum samples tested by HI and highest titer for each swIAV subtype within pig categories.

Pig Category	Piglets 1–4 Weeks	Piglets 5–10 Weeks	Piglets 11–20 Weeks	Fatteners ˃20 Weeks	Gilts	First Parity Sows	Sows	Boars	Pigs of Unknown Category
*n* examined samples	8	38	13	91	74	43	62	19	62
IAV subtypes	IAV positive (highest Ab titre)								
H1N1	6 (1:16)	14 (1:64)	4 (1:32)	22 (1:128)	41 (1:128)	20 (1:512)	40 (1:512)	9 (1:256)	22 (1:16)
H3N2	7 (1:64)	18 (1:128)	4 (1:512)	9 (1:128)	45 (1:128)	30 (1:2048)	51 (1:512)	2 (1:128)	9 (1:16)
H1N2	5 (1:32)	16 (1:128)	4 (1:32)	8 (1:128)	31 (1:128)	20 (1:512)	14 (1:512)	1 (1:128)	9 (1:16)
H1N1 + H3N2	1	0	1	1	25	6	19	1	1
H3N2 + H1N2	0	5	1	0	8	4	8	0	1
H1N1 + H1N2	0	1	2	0	5	0	0	0	4
H1N1 + H3N2 + H1N2	5	11	1	6	7	13	9	1	3
Negative	1	15	7	65	5	10	8	14	34

## Data Availability

Data used for the review is available upon reasonable request to the corresponding author.

## Supplementary Materials

The following are available online at https://www.mdpi.com/article/10.3390/pathogens10111527/s1, Figure S1: MDCK cell culture, 10×. Inoculated sample 2319/4/2011 (H1N1). (a) Early formation of influenza-induced CPE, 2nd day postseed. (b) Influenza-induced CPE, 4th day postseed. Infected cells are detached and floating in the culture medium. Table S1: Results of serological testing (ELISA) within different pig categories. Table S2: Mean seroprevalence of IAV positive serum samples subtyped by HI (%) presented in different age groups. Table S3: Total of lung samples tested by Real-time RT-qPCR (M gene) and quantification cycle threshold (Cq) values. Table S4. Total of nasopharyngeal swabs tested by Real-time RT-qPCR (M gene) and quantification cycle threshold (Cq) values.
