# Supplementary material for: Improving Current Knowledge on Seroprevalence and Genetic Characterization of Swine Influenza Virus in Croatian Pig Farms: A Retrospective Study
Source: Pathogens. 2021 Nov 22;10(11):1527. doi: 10.3390/pathogens10111527 (PMC8623915; doi:10.3390/pathogens10111527)
Supplement: Supplementary file 1 [file pathogens-10-01527-s001.zip › pathogens-1423647-supplementary.pdf]

Table S1. Results of serological testing (ELISA) within different pig categories.

Category		*n* examined samples	IAV NP Ab positive	
			n	%
Piglets	1-4 weeks	35	8	22,9
	5-10 weeks	260	33	12,7
	11-20 weeks	249	27	10,8
Fatteners >20 weeks		153	86	56,2
Gilts		278	99	35,6
First parity sows		70	43	61,4
Sows		130	64	49,2
Boars		132	17	12,9
Pigs of unknown category		229	89	38,9
Total:		1536	466	30,3%

Table S2. Mean seroprevalence of IAV positive serum samples subtyped by HI (%) presented in different age groups.

**category**	**piglets 1-4 weeks**	**piglets 5-10 weeks**	**piglets 11-20 weeks**	**fatteners >20 weeks**	**gilts**	**first parity sows**	**sows**	**boars**	**pigs of unknown category**
**tested sera samples (*n*)**	8	38	13	91	74	43	62	19	62
**HI (%)**									
**H1N1**	75	36.8	30.8	24.2	55.4	46.5	64.5	47.4	35.5
**H3N2**	87.5	47.4	30.8	9.9	60.8	69.8	82.3	10.5	14.5
**H1N2**	62.5	42.1	30.8	8.8	41.9	46.5	22.6	5.3	14.5
**negative**	12.5	39.5	53.8	71.4	6.8	23.3	12.9	73.7	54.8

Table S3. Total of lung samples tested by Real-time RT-qPCR (M gene) and quantification cycle threshold (Cq) values.

**County**	**Farm (F)/ backyard farm (BF)/slaughterhouse (SL)**	***n* lungs**	**Cathegory**	**Sample ID**	**Ct**
Brod-Posavina	BF	3	Piglets 10-12 weeks	2209/1-3/2014	no Ct
	BF	2	Piglets 10-12 weeks	631/2/2015	no Ct
				631/3/2015	**28,61**
	BF	3	Piglets 9-12 weeks	P1-3/2015	no Ct
Varaždin	BF	4	Piglets 4-10 weeks	972/1-4/2014	no Ct
Vukovar-Srijem	F14	4	Piglets 9-14 weeks	2319/1/2011	-
				2319/2/2011	24,40
				2319/3/2011	30,26
				2319/4/2011	19,32
		5	Piglets 9-14 weeks	1600/1/2012	38,41
				1600/9/2012	23,12
				1600/1,3,10/2012	-
		7	Piglets 4-10 weeks	331/1-7/2016	-
	F13	3	Piglets 4-6 weeks	1954/1-3/2013	-
		3	Piglets 3 weeks	PPA1-3/2014	-
		6	Weaners	PA1-6/2015	-
		3	Piglets 4 weeks	PA7-9/2015	-
		3	Piglets 4-6 weeks	853/1-3/2016	-
		5	Piglets 4-10 weeks	854/1/2016	16,94
				854/5/2016	22,94
				854/2,3,4/2016	-
		4	Piglets 4-10 weeks	855/1-3/2016	-
				855/4/2016	32,28
	F18	3	Piglets 9-12 weeks	2571/1-3/2013	-
Zagreb	SL	72	Fatteners	1-72/2015	-

	F16	4	Piglets 6-11 weeks	2500/1-4/2012	-
		6	Piglets 6-11 weeks	2318/1-6/2013	-
		1	Piglet 10 weeks	1002/2014	-
		1	Piglet 4 weeks	PP/2015	-
**Total:**		**142**		**9 positive**	

Table S4. Total of nasopharyngeal swabs tested by Real-time RT-qPCR (M gene) and quantification cycle threshold (Cq) values.

County	Farm (F)/ backyard farm (BF)/slaughterhouse (SL)	*n* lungs	Cathegory	Sample ID	Cq
Vukovar-Srijem	F14	10	Sows	B1-10/2012	-
		10	Piglets 6-11 weeks	B11-20/2012	-
	F13	9	Piglets 4-6 weeks	1/2016	26,82
				2/2016	32,71
				3/2016	23,96
				4/2016	37,46
				5/2016	32,10
				6/2016	35,74
				7/2016	37,24
				8/2016	-
				9/2016	-
**Total:**		**29**		**7 positive**	

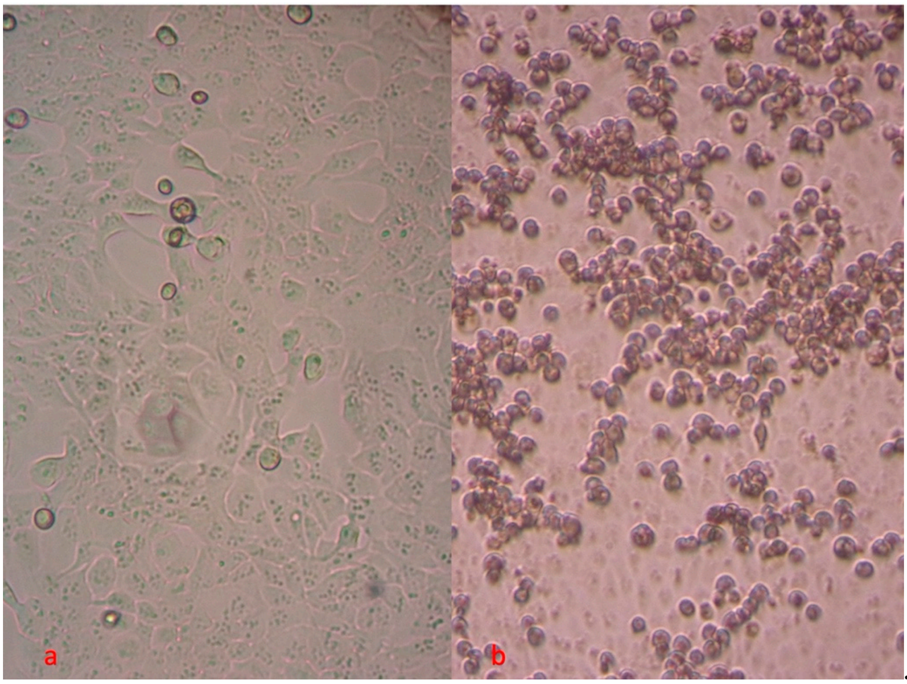

Figure S1. MDCK cell culture, 10x. Inoculated sample 2319/4/2011 (H1N1). ***(a)*** Early formation of influenza-induced CPE, 2nd day postseed. ***(b)*** Influenza-induced CPE, 4th day postseed. Infected cells are detached and floating in the culture medium.
